# COVID-19 stressors and mental health problems amongst women who arrived as refugees and those born in Australia

**DOI:** 10.1371/journal.pgph.0002073

**Published:** 2023-07-03

**Authors:** Susan J. Rees, Mohammed Mohsin, Alvin Kuowei Tay, Batool Moussa, Louis Klein, Nawal Nadar, Fatima Hussain, Yalini Krishna, Batoul Khalil, Mariam Yousif, Derrick Silove, Jane Fisher

**Affiliations:** 1 Faculty of Medicine, Discipline of Psychiatry, School of Clinical Medicine, University of New South Wales, Sydney, Australia; 2 Mental Health Research Unit, Liverpool Hospital, SWSLHD, New South Wales Health, Liverpool, Australia; 3 Division of Social Sciences in Public Health and Preventive Medicine, Global and Women’s Health, Monash University, Melbourne, Victoria, Australia; Southern Cross University, AUSTRALIA

## Abstract

Women from refugee background residing in high income countries are at greater mental health risk during the COVID-19 pandemic given their higher baseline prevalence of mental disorders, trauma exposures and social adversities. During the COVID-19 pandemic we drew on data from wave-4 of the WATCH cohort study, collected between October 2019 and June 2021. We conducted a cross-sectional analysis to compare the prevalence of common mental disorders (CMDs) from the sample of 650 consecutively recruited women, 339 (52.2%) from the refugee-background who were resettled in Australia and 311 (48.8%) randomly and contemporaneously selected Australian born women. We assessed COVID-19 psychosocial stressors: 1. COVID-related material hardship and 2. COVID-related fear and stress. We examined for associations between scores on these two items and CMDs in each group respectively. Compared to Australian-born woman, women from refugee background recorded a significantly higher prevalence of Major Depressive Disorder (MDD) (19.8% vs 13.5%), PTSD (9.7% vs 5.1%), Separation Anxiety Disorder (SEPAD) (19.8% vs 13.5%) and Persistent Complicated Bereavement Disorder (PCBD) (6.5% vs 2.9%). In refugee women, associations were found between COVID-related material hardship and CMDs [MDD, Relative Risk (RR) = 1.39, 95%CI: 1.02–1.89, p = 0.02] as well as between COVID-related fear and stress and CMDs (MDD, RR = 1.74, 95%CI: 1.04–2.90, p = 0.02 p = 0.02). For Australian-born women, associations were more commonly found between CMDs and material hardship. Our study demonstrates that both women from refugee background and those born in Australia are experiencing significant rates of CMD during the pandemic and that material hardship is an associated factor. We found that women from refugee background are at greater risk for mental health problems and are more likely to report an association of those problems with fear and stress related to COVID_19. All women, and particularly those from refugee background, require urgent and specialised attention to their mental health and psychosocial problems during this pandemic.

## Introduction

Women from refugee backgrounds (“refugee women”) living in western countries may be experiencing heightened psychological stress during the COVID pandemic because of their pre-existing vulnerabilities arising from exposure to traumatic events, their high baseline level of common mental disorders (CMDs), and the unique psychosocial challenges they confront during the resettlement process.

The predominant research examining COVID-related stressors on mental health derives from general populations in high income countries. These studies show associations between exposure to COVID-19 pandemic stressors and reported psychiatric symptoms including depression and anxiety [1–4]. Cross-sectional associational studies have been supported by longitudinal evidence suggesting that prior exposure to COVID-19 stressors increases later onset or exacerbation of CMDs and distress [5–10]. Nevertheless, a meta-analysis suggested that these associations were more pronounced during the early rather than the later phases of the pandemic [11]. Importantly, a UK study found that socially disadvantaged subpopulations, for example, those with lower incomes, experienced more adverse mental health impact from exposure to the pandemic, and that social and cultural histories and personal experiences of COVID-19 were important in determining relative psychiatric risk [12].

Although they are of considerable demographic size and have unique mental health profiles, there are no large COVID-19-specific mental health studies of women who settle in high income countries from refugee backgrounds. The few studies to date indicating a higher mental health burden in a similar population have been limited by convenience sampling, or did not differentiate between refugees and other migrants [13–17]. One Australian study used an online convenience sample, finding a significant impact of COVID-19 on the mental health of people from refugee background [18]. Another online self-report survey found that mental health across a mixed group of refugees and migrants during the COVID-19 pandemic was significantly impacted, particularly among those with insecure housing, older respondents, and women [19].

Women’s mental health may be at particular risk during COVID-19 because of factors unique to their gender, including a higher vulnerability to CMDs in general, the impact of pregnancy and early child-rearing, greater socio-economic deprivation, and significantly higher exposure to traumatic events [20,21]. All these factors may exacerbate the impact of COVID-19 related stressors including women’s greater representation in insecure employment and having a disproportionate responsibility for domestic and caring tasks [13,22,23]. An intersectional approach highlights the multiple intersecting contextual and psychological inequalities that can further heighten the specific risks of CMDs for women from refugee background [24]. Our recently published commentary, written to guide mental health practitioners during the pandemic, considered specific mental health risks during COVID-19 for people from refugee background living in high income countries, including exposure to prior trauma, distrust of authorities, fear for family in the home country, and current financial adversity [24]. The paucity of empirical knowledge in the field is particularly concerning in the context of the higher pre-COVID risk for trauma-related mental disorders amongst people from refugee background [20,25–31].

Psychosocial risks to mental health during COVID-19 that have been measured in general populations can be categorised into those that are *extrinsic* in nature, usually concerning material hardship, and those that are *intrinsic* in nature, mostly concerning psychological fear and stress related to COVID-19 disease or conditions [18,24]. In other studies, hardship is commonly related to financial problems, being compelled to remain under lockdown conditions, and living with unpredictable policies and changes in public health advice. Psychological fear and stress on the other hand are often associated with concerns about personal health status and illness from COVID-19, worry about the health and wellbeing of family members, and isolation from family and friends [16].

The overall aim of the present study was to examine associations of COVID-19 related material hardship and COVID-19 related fear and stress with a range of CMDs amongst refugee women resettled in Australia. We included data from a sample of Australian-born women who were assessed in parallel with the refugee women to examine areas of commonality and difference between the two groups in relation to the associations between the two COVID-19 stressor domains (material and fear and stress) and key mental disorder outcomes.

We first aimed to identify the level of self-rated stress reported by both groups of women on two items assessing the severity of COVID-19 related material hardship and COVID-19 related fear and stress. We also assessed the concurrent prevalence of theoretically relevant CMDs including Major Depressive Disorder (MDD), Post-Traumatic Stress Disorder (PTSD), Persistent Complicated Bereavement Disorder (PCBD), panic disorder (PD), and Separation Anxiety Disorder (SEPAD). The inclusion of these specific disorders was based on assessment of prior relevant literature and our extensive scoping work with communities and leaders in the refugee field. We did not inquire about alcohol and substance use, which was assumed to be very low, and the topic can be confronting on cultural and religious grounds. The core data used in our analyses are derived from a current larger Australian-based cohort study, with the addition of two COVID-19 related indices [20].

We hypothesised that refugee women would record a higher prevalence of all CMDs compared to Australian-born women and that CMDs would be associated with more severe COVID-19 related material hardship and COVID-19 related fear and stress in the refugee group. We further hypothesised that exposure to past trauma would be associated with higher levels of COVID-19 related material hardship and COVID-19 related fear and stress in refugee and Australian born women.

## Materials and methods

### Ethics and research personnel

The study was approved by the South Western Sydney Local Health District Human Research Ethics Committee (HC13049) and Monash Health Ethics Committee, Australia. Participants were provided with information about the study, and those electing to participate signed written consent forms and were remunerated for their time. Eight women field workers from matching language backgrounds were given extensive training consisting of three formal training days followed by tests of competence [20]. Training covered research methods and practice, sensitive interviewing techniques, and use of the diagnostic mental health and World Health Organization (WHO) measures. Staff received ongoing support, monitoring, and supervision during the study. This study followed Strengthening the Reporting of Observational Studies in Epidemiology (STROBE) reporting guidelines. The study protocol is published [21].

### Participants

The present study compares CMDs and COVID-19 stressors amongst women from refugee background and those who were Australian born. We undertook this cross-sectional study, utilising data from the fourth wave of data from the WATCH cohort study of systematically recruited women of child-bearing age born in Australia and women from refugee background [20]. The wave-4 data coincided with the COVID-19 period and was collected between October 2019 and June 2021. Australia was affected by the COVID-19 pandemic from February 2020 onwards, so of 775 eligible for wave-4, 650 women were able to participate in this study because of their exposure to the COVID-19 pandemic at wave-4. Of those who participated 311 (48.8%) were Australia-born and 339 (52.2%) were women from refugee backgrounds.

### Recruitment for the WATCH cohort study

This cohort was originally recruited between January 2015 and March 2016. The recruitment of participants into the cohort study, including those born in Australia and from refugee background, occurred at the same time and from the same three public antenatal clinics; two in the city of Sydney, New South Wales, Australia, and one in the city of Melbourne, Victoria, Australia [20]. Recruitment occurred at a woman’s first appointment at the clinic, which most commonly occurred between 12 and 20 weeks gestation. Women with overt psychosis, severe medical illness, and obvious intellectual impairment were excluded. Consecutive sampling was used to systematically recruit women from conflict-affected countries. The consecutive sampling strategy (approaching every patient who met the selection criteria over the specified period) assisted with ensuring the cohort was representative of the target population and the findings therefore more generalisable to the population of women. This sampling method is effective when the target population is small, however with larger populations it can produce an excessively large sample (hence we used randomised sampling for the Australian born women, discussed below).

The recruitment of women from refugee background included all conflict-affected Arabic-speaking countries, Sudan, and Sri Lanka (Tamil-speaking) [20,21]. These nations represented the largest intake groups from conflict-affected regions entering Australia and other high-income countries at the time of this study. By limiting the study to these language groups, we sought to contain both the problems of transcultural measurement error and small cell sizes. Country of origin was identified by searching all clinic records for upcoming appointments. An Arabic speaking cross-cultural expert was tasked to identify the participants from refugee background who would be approached. She searched all clinic lists for either requests for an interpreter, or culturally recognisable surnames. Country of birth data were also checked against clinic appointment lists. Women members of the research team who spoke the same language as eligible women approached those identified women in the waiting room and, following consent, conducted interviews.

Because women born in Australia attended the clinics in substantially larger numbers than those from conflict-affected countries we elected to undertake a parallel sampling strategy over a similar time frame. With that larger cohort we applied a computer-generated randomisation procedure to identify daily a subset of women born in Australia. The randomised procedure was based on a kish grid, with the primary number being determined by the total of attendees listed to attend the clinic on each day (each arrival being allocated a number).

### Survey measures

#### Cultural accuracy

All instruments were selected based on their previous psychometric evaluations and use across cultures. Translations of instruments were subjected to systematic monitoring of cultural and linguistic accuracy in the study’s languages using the Translation Monitoring form approach [32,33]. After translation and back-translation procedures were performed, and final refinements were made by groups of linguistic experts.

#### Socio-demographic characteristics

Socio-demographic measures were adopted from the Australian Bureau of Statistics National Census items including: place of usual residence, age, marital status, housing status, highest level of educational attainment and employment status [34]. We measured access to social support during COVID-19 by inquiring ‘number of friends and family members you can rely on for serious problems.’ We excluded in this analysis a measure for the length of time in Australia because earlier analysis had shown that, unlike level of education or employment status, it was not significantly associated with any of the mental health indicators [20].

#### Traumatic events

We assessed lifetime exposure to traumatic events (TEs) based on the inventory used in the World Mental Health Survey [35,36] which we modified for the experiences of the refugee and Australian born participants [37–39]. We adapted a list of 19 traumatic items including political imprisonment, assault, torture, witnessing murder, exposure to atrocities, losses/separations of family or close others, abuse as a child, any seriously traumatic or life-threatening event, and deprivation of medical care for self or others in situations of severe illness. Events were recorded as lifetime exposure (ever exposed 1; never exposed 0); and a total trauma count was generated based on the number of events endorsed (ranges 0 to 11) [40]. Because of sample size distributions in each count, for statistical analysis we arbitrarily grouped total TE counts into 5 higher ordered categories (0, 1, 2, 3–4, 5 and more).

#### Finance-related stressors

Finance-related stressors comprised of seven items relating to difficulties such as paying bills and affording enough food and heating (each items coded as ‘1’ for ‘yes’ and ‘0’ for ‘no’). A composite index for a total number of finance-related stressors was generated based on the number of stressors endorsed (score range, 0–7). Because of sample size distributions in each count, for statistical analysis we arbitrarily grouped total number of stressors into three higher ordered categories (0, 1–2, 3 and more).

#### Mental health measures

We used the Mini-International Neuropsychiatric Interview (MINI) based on the Diagnostic and Statistical Manual of Mental Disorders (Fourth Edition) (DSM-IV) [41,42] to assess major depressive disorder (MDD) and other common mental health measures including Post-Traumatic Stress Disorder (PTSD), Persistent Complicated Bereavement Disorder (PCBD), Panic Disorder (PD), and Separation Anxiety Disorder (SEPAD). We selected DSM-IV in preference to DSM-5 because the latter had not yet been used extensively across cultures at the commencement of the cohort study. The MDD measure consists of 9 items assessing depression symptoms in the last two weeks. Women who answered yes for either item 1 or 2; and answered yes for at least 3 of other items were classified as having MDD. The PTSD interview consists of 15 items assessing PTSD symptoms in the last month. Women who answered yes for items ‘1 to 3’; and answered yes at least for 3 items of ‘4 to 10’; and answered yes for 2 of the remaining 5 items were classified as having PTSD. The PD measure consists of 17 items and women who answered yes at least for 4 of 17 items were classified as having panic disorder. The PCBD measure consists of 12 items and women who answered yes for items ‘1 to 3’; and answered yes at least for 5 of the items ‘4 to 12’ were classified as having PCBD. The SEPAD measure consists of 15 items and women who answered yes at least for 3 of 15 items were classified as having SEPAD.

#### COVID-19 related psychosocial problems

We identified and included two broad questions to examine COVID-19 related psychosocial problems. These questions have face-validity given they were developed from existing knowledge of the aetiology of problems identified in our previous paper, and the prevailing literature on COVID-19 psychosocial problems [4–6,22]. The items included: (1) Material hardship related to COVID-19 (referring to economic and related social challenges); and (2) Fear or stress associated with COVID-19 (referring to emotional and psychological challenges). The COVID-19 related items were rated on a 3-point Likert scale (0 = no problem at all, 1 = a problem, 2 = a very serious problem). To support our statistical analysis, the answers for each of the COVID-19 items were recoded into two groups: 0 = ‘no problem at all’, 1 = ‘a problem or a very serious problem’. Due to the distinct nature of these items, statistical analysis for each were carried out separately.

### Statistical analysis

Descriptive statistics were generated for key sociodemographic variables including age, highest level of educational attainment, women’s employment, men’s (partners) employment, number of friends or family members who can be relied on for serious problems, ever been homeless, number of traumatic events exposures and number of finance-related stressors. We also present the descriptive statistics for the two COVID specific hardship measures; and prevalence of CMDs for refugee and Australian women. Using bivariate (cross-tabular) analyses, we examined the potential risk factors associated with COVID specific material hardships and fear and stress for each group of women respectively. We implemented *χ*^2^test to examine the preliminary bivariate associations of all sociodemographic variables, access to social support during the pandemic, and past trauma exposure with each of the two COVID-19 related problems (% of ‘a problem/a very serious problem’) for both Australian born and women from refugee background respectively. Results of the bivariate analyses are represented as percentages. Then we incorporated the significant predictors into the multivariable logistic regression model, noting that predictor variables with high levels of multi-collinearity were eliminated by default in the regression model.

As the two COVID specific measures comprise dichotomous variables (0 = ‘no problem at all’, 1 = ‘a problem or a very serious problem’), multiple log-binomial regression analyses were used to examine the relative contributions of each statistically significant (p < .05) predictor variable with an associated risk of any COVID-19 related problem [43]. Using the combined sample of Australian born and women from refugee background, two multiple log-binomial regression analyses were performed for the two COVID-19 specific measures. In addition to all potential predictors, multiple log-binomial regression models were adjusted for two groups of sampled women: Australian born women (coded as ‘0’; assigned as reference category) and women from refugee background (coded as ‘1’). Adjusted relative risk ratios (aRRs) with 95% confidence intervals (95% CI) are provided. Finally, we also examined the impact of the two COVID-19 related problems on common mental disorders including: Major Depressive Disorder (MDD), Post-Traumatic Stress Disorder (PTSD), Persistent Complicated Bereavement Disorder (PCBD), Panic Disorder (PD), and Separation Anxiety Disorder (SEPAD). Statistical analyses were performed by using IBM SPSS version 27 [44].

## Results

### Socio-demographic characteristics

Of the 650 study women, 339 (52.2%) had migrated to Australia from conflict-affected countries (women from refugee backgrounds) and 311 (47.8%) were born in Australia. The most highly represented countries of origin in the women from refugee backgrounds were Iraq (n = 117; 34.50%), Lebanon (n = 59; 17.4%), Sri Lanka (n = 51; 15.0%) and Sudan (n = 25; 7.4). The key sociodemographic characteristics of the women from refugee backgrounds and Australian born women are presented in Table 1. The mean age was 34.0 (SD = 5.6) years for women from refugee backgrounds and for Australian born women was 33.5 (SD = 5.5) years. Almost half (50.6%) of the refugee background and 54.0% of Australian born women were aged between 25–34 years; and less than 5% in both groups were aged under 25 years.

**Table 1:** Percentage distribution (95% CI) of Australian born and refugee background participants who completed COVID-19 questionnaires by socio-demographic characteristics.

Socio-demographic characteristics	Australian born women (n = 311)		^a^ Women from Refugee Background (n = 339)	
	Number of women	% (95% CI)	Number of women	% (95% CI)
^***#***^ ***All***	*311*	*100*.*0*	*339*	*100*.*0*
**Age (in years)**				
<25 years	13	4.2 (2.5–7.0)	9	2.7 (1.4–5.0)
25–34 years	168	54.0 (48.5–59.5)	171	50.4 (45.1–55.7)
35 years and above)	130	41.8 (36.5–47.3)	159	46.9 (41.7–52.2)
*Mean age (SD)[95% CI]*		*33*.*5 (5*.*3) [32*.*7–34*.*3]*		*34*.*0 (5*.*6)[33*.*2–34*.*8]*
**Highest level of educational attainment**				
No post school qualification	107	34.5 (29.4–40.0)	129	38.6 (33.6–43.9)
Diploma and vocational education	97	31.3 (26.4–36.7)	89	26.6 (22.2–31.6)
University degree	106	34.2 (29.1–39.6)	116	34.7 (29.8–40.0)
**Ever been Homeless**				
No	297	95.5 (92.6–97.3)	316	93.5 (0.3–95.7)
Yes	14	4.5 (2.7–7.4)	22	6.5(4.3–9.7)
**Women’s Employment**				
Employed	175	56.3 (50.7–61.7)	106	31.3 (26.6–36.4)
Unemployed	136	43.7 (38.3–49.3)	233	68.7 (63.6–73.4)
**Men’s Employment**				
Employed	250	86.8 (82.4–90.2)	248	74.5 (69.5–78.9)
Unemployed	38	13.2 (9.8–17.6)	85	25.5 (21.1–30.5)
**Number of traumatic events exposures**				
None	127	40.8 (35.5–46.4)	67	19.9 (16.0–24.5)
1	104	33.4 (28.4–38.9)	70	20.8 (16.8–25.4)
2	46	14.8 (11.3–19.2)	76	22.6 (18.4–27.3)
3–4	24	7.7 (5.2–11.2)	68	20.2 (16.2–24.8)
5 and more	10	3.2 (1.8–5.8)	56	16.6 (13.0–21.0)
***Mean (SD)*** *[95% CI]*		*1*.*0 (1*.*2)[0*.*8–1*.*2]*		*2*.*3 (2*.*1)[2*.*0–2*.*6]*
^**b**^ **No. of friends/family members who can be relied on for serious problems**				
> = 5	202	65.0 (59.5–70.0)	88	26.1 (21.7–31.1)
3–4	70	22.5 (18.2–27.5)	93	27.6 (23.1–32.6)
< = 2	39	12.5 (9.3–16.7)	156	46.3 (41.0–51.6)
**No. of finance related stressors**				
0	247	79.4 (74.6–83.5)	220	64.9 (59.7–69.8)
1–2	31	10.0 (7.1–13.8)	55	16.2 (12.7–20.5)
3 and more	33	10.6 (7.7–14.5)	64	18.9 (15.1–23.4)
***Mean (SD)*** *[95% CI]*		*0*.*6 (1*.*3)[0*.*4–0*.*8]*		*1*.*0 (1*.*5)[0*.*8–1*.*2]*

**Note:** *SD*, Standard deviation; *NA*, Not applicable.

^**a**^ Country of birth for women from refugee background No. (%): Iraq 117 (34.5%); Lebanon 59 (17.4%); Sudan 25 (7.4%).

Syria 17 (5.0%); Egypt 15 (4.4%); Afghan 6 (1.8%); Sri Lanka 51 (15.0%); Others 49 (14.5%).

^**b**^ Number of friends/family participants can rely on for serious problems computed based on two items: 1) Number of family members can rely on for serious problems? 2) Number of friends can rely on for serious problems?

^***#***^*Numbers do not always add up to 311 or 339 due to exclusion of not stated or missing cases*.

One third of the women in both groups held a university degree (34.2% vs. 34.7%). A small proportion (n = 14; 4.5%) of the Australian born women reported that they were homeless at some point of their life and this rate was slightly higher (n = 22; 6.5%) for women from refugee background. Over half (56.3%) of the Australian born women were employed and this rate was lower (31.3%) for women from refugee background. Most of the partners for both Australian born and women from refugee background were employed. Women from refugee background were more likely to have experienced one or more traumatic event. Australian born women reported higher levels of family support which they could rely on for serious problems (Table 1). Almost a third (35.1%) of the refugee background women had reported at least one or more finance-related stressors and this rate was 20.6% for Australian born women. Of the refugee-specific characteristic items, almost half of the women had migrated to Australia after 2010; and approximately one third reported low adaptation to the Australian way of doing things (Table 1).

### COVID-19 specific psychosocial measures

Distribution of the participants by the two COVID-19 related measures for women from refugee backgrounds and Australian born women are presented in Table 2. A third (32.5%) of the Australian born women reported having ‘a problem or a very serious problem’ associated with material hardship related to COVID-19; and this rate was higher (47.2%) for women from refugee backgrounds. Nearly half (47.3%) of the Australian born women reported having ‘a problem or a very serious problem’ with fear or stress associated with COVID-19; and this rate was significantly higher (68.7%) for women from refugee backgrounds (Table 2).

**Table 2:** Percentage distribution (95% CI) for Australian born and refugee background participants by COVID-19 related measures.

COVID-19 related measures	Australian born women (n = 311)		Women from Refugee Background (n = 339)	
	Number of women	% (95% CI)	Number of women	% (95% CI)
^***#***^ ***All***	*311*	*100*.*0*	*339*	*100*.*0*
**Hardship related to COVID-19**				
No problem at all	210	67.5 (62.1–72.5)	179	52.8 (47.5–58.0)
A problem	83	26.7 (22.1–31.9)	105	31.0 (26.3–36.1)
A very serious problem	18	5.8 (3.7–8.9)	55	16.2 (12.7–20.5)
***A problem/a very serious problem***	***101***	***32*.*5 (27*.*5–37*.*9)***	***160***	***47*.*2 (41*.*9–52*.*5)***
**Fear or stress associated with COVID-19**				
No problem at all	164	52.7 (47.2–58.2)	106	31.3 (26.7–36.4)
A problem	121	38.9 (33.7–44.4)	162	47.8 (42.5–53.1)
A very serious problem	26	8.4 (5.8–11.9)	71	20.9 (16.9–25.6)
***A problem/a very serious problem)***	***147***	***47*.*3 (41*.*8–52*.*8)***	***233***	***68*.*7 (63*.*6–73*.*4)***

^***#***^*Numbers do not always add up to 311 or 339 due to exclusion of not stated or missing cases*.

### Putative predictors of COVID-19 related measures: Bivariate analysis

#### Material hardship related to COVID-19

Bivariate analyses showed that for women from refugee backgrounds, age (p = 0.047), educational level (p = 0.019) and higher number of traumatic event exposures (p = 0.017) were significantly associated with greater material hardship problems related to COVID-19. For Australian born women, husband/partners unemployment (p = 0.010) and higher number of finance related stressors (p = 0.003) was significantly associated with greater material hardship related to COVID-19 (Table 3A).

**Table 3:** a. Association of socio-demographic characteristics with percentage of Australian Born and Refugee Background participants who reported ‘a problem/a very serious problem’ for ‘Hardship related to COVID-19’. **b**. Association of socio-demographic characteristics with percentage of women who reported ‘a problem/a very serious problem’ related to ‘Fear or stress associated with COVID-19’ for Australian Born and Women from Refugee Background.

	Hardship related to COVID-19			
	Australian born women		Women from Refugee Background	
Socio-demographic characteristics	Number	A problem/a very serious problem: row % (n)	Number	A problem/a very serious problem: row % (n)
** All**	**311**	**32.5 (101)**	**339**	**47.2 (160)**
**Age (in years)**				
<25 years	13	15.4 (2)	9	44.4 (4)
25–34 years	168	33.9 (57)	171	53.8 (92)
35 years and above)	130	32.3 (42)	159	40.3 (64)
*Test statistic χ*^*2*^*(df) and p values* ^**b**^		*χ*^*2*^*(2) = 1*.*89; 0*.*388*		*χ*^*2*^*(2) = 6*.*10; 0*.*047*
**Highest level of educational attainment**				
No post school qualification	107	38.3 (41)	129	38.0 (49)
Diploma and vocational education	97	32.0 (31)	89	57.3 (51)
University degree	106	27.4 (29)	116	47.4 (55)
*Test statistic χ* ^*2*^ *(df) and p-values*		*χ*^*2*^*(2) = 2*.*93; 0*.*230*		*χ*^*2*^*(2) = 7*.*97;* ***0*.*019***
**Ever been Homeless**				
No	297	31.6 (94)	316	45.9 (145)
Yes	14	50.0 (07)	22	63.6 (14)
*Test statistic χ* ^*2*^ *(df) and p-values*		*χ*^*2*^*(1) = 2*.*05; 0*.*157*		*χ*^*2*^*(1) = 2*.*60; 0*.*125*
**Women’s Employment**				
Employed	175	29.7 (52)	106	50.9 (54)
Unemployed	136	36.0 (49)	233	45.5 (106)
*Test statistic χ* ^*2*^ *(df) and p-values*		*χ*^*2*^*(1) = 1*.*39; 0*.*238*		*χ*^*2*^*(1) = 0*.*868; 0*.*351*
** Men’s Employment**				
Employed	250	29.2 (73)	248	47.6 (118)
Unemployed	38	50.0 (19)	85	47.1 (40)
*Test statistic χ* ^*2*^ *(df) and p-values*		*χ*^*2*^*(1) = 6*.*56;* ***0*.*010***		*χ*^*2*^*(1) = 0*.*01; 0*.*934*
**Number of traumatic events exposures**				
None	127	27.6 (35)	67	35.8 (24)
1	104	35.6 (37)	70	44.3 (31)
2	46	30.4 (14)	76	42.1 (32)
3–4	24	37.5 (09)	68	63.2 (43)
5 and more	10	60.0 (06)	56	51.8 (29)
*Test statistic χ* ^*2*^ *(df) and p-values*		*χ*^*2*^*(4) = 5*.*67; 0*.*225*		*χ*^*2*^*(4) = 12*.*00;* ***0*.*017***
**No. of friends/family members can be relied on for serious problems**				
> = 5	202	32.2 (65)	88	52.3. (46)
3–4	70	34.3 (24)	93	47.3 (44)
< = 2	39	30.8 (12)	156	43.6 (68)
*Test statistic χ* ^*2*^ *(df) and p-values*		*χ*^*2*^*(2) = 0*.*17; 0*.*921*		*χ*^*2*^*(2) = 1*.*71; 0*.*425*
**No. of finance related stressors**				
0	247	28.3 (70)	220	36.8 (81)
1–2	31	38.7 (12)	55	54.5 (30)
3 and more	33	57.6 (19)	64	76.6 (49)
*Test statistic χ* ^*2*^ *(df) and p-values*		*χ*^*2*^*(2) = 11*.*96;* ***0*.*003***		*χ*^*2*^*(2) = 32*.*84;* ***0*.*001***

	**Fear or stress associated with COVID-19**			
	**Australian born women**		**Women from Refugee Background**	
**Socio-demographic characteristics**	Number	A problem/a very serious problem: row % (n)	Number	A problem/a very serious problem: row % (n)
** All**	**311**	**47.3 (147)**	**339**	**68.7 (233)**
**Age (in years)**				
<25 years	13	46.2 (6)	9	55.6 (5)
25–34 years	168	44.0 (74)	171	70.2 (120)
35 years and above)	130	51.5 (67)	159	67.9 (108)
*Test statistic χ*^*2*^*(df) and p values* ^**b**^		*χ*^*2*^*(2) = 1*.*65; 0*.*437*		*χ*^*2*^*(2) = 0*.*94; 0*.*625*
**Highest level of educational attainment**				
No post school qualification	107	44.9 (48)	129	65.9 (85)
Diploma and vocational education	97	49.5 (48)	89	70.8 (63)
University degree	106	48.1 (51)	116	69.0 (80)
*Test statistic χ* ^*2*^ *(df) and p-values*		*χ*^*2*^*(2) = 0*.*47; 0*.*792*		*χ*^*2*^*(2) = 0*.*62; 0*.*732*
**Ever been Homeless**				
No	297	47.8 (142)	316	67.4(213)
Yes	14	35.7 (5)	22	86.4 (19)
*Test statistic χ* ^*2*^ *(df) and p-values*		*χ*^*2*^*(1) = 0*.*78; 0*.*376*		*χ*^*2*^*(1) = 3*.*43; 0*.*106*
**Women’s Employment**				
Employed	175	50.3 (88)	106	69.5 (73)
Unemployed	136	43.4 (55)	233	68.4 (160)
*Chi-square test*: *p values*		*χ*^*2*^*(1) = 0*.*46; 0*.*226*		*χ*^*2*^*(1) = 0*.*44; 0*.*833*
**Men’s Employment**				
Employed	250	45.6 (114)	248	68.1 (169)
Unemployed	38	44.7 (17)	85	71.8 (61)
*Test statistic χ* ^*2*^ *(df) and p-values*		*χ*^*2*^*(1) = 0*.*01;* 0.921		*χ*^*2*^*(1) = 0*.*39;* 0.533
**Number of traumatic events exposures**				
None	127	35.4 (45)	67	55.2 (37)
1	104	56.7 (59)	70	61.4 (43)
2	46	54.3 (25)	76	72.7 (56)
3–4	24	45.8 (11)	68	83.6 (56)
5 and more	10	70.0 (07)	56	71.4 (40)
*Test statistic χ* ^*2*^ *(df) and p-values*		*χ*^*2*^*(4) = 13*.*89;* ***0*.*008***		*χ*^*2*^*(4) = 15*.*09;* ***0*.*005***
**No. of friends/family members can be relied on for serious problems**				
> = 5	202	47.5 (96)	88	71.9 (64)
3–4	70	48.6 (34)	93	65.6 (61)
< = 2	39	43.6 (17)	156	68.4 (106)
*Test statistic χ* ^*2*^ *(df) and p-values*		*χ*^*2*^*(2) = 0*.*26; 0*.*876*		*χ*^*2*^*(2) = 0*.*85; 0*.*655*
**No. of finance related stressors**				
0	247	44.9 (111)	220	62.4 (138)
1–2	31	61.3 (19)	55	70.9 (39)
3 and more	33	51.5 (17)	64	88.9 (56)
*Test statistic χ* ^*2*^ *(df) and p-values*		*χ*^*2*^*(2) = 3*.*22; 0*.*200*		*χ*^*2*^*(2) = 16*.*01;* ***0*.*001***

**Note:** *NA*, Not applicable.

^**b**^ *Chi-square test* conducted to examine the significant variation of ‘COVID-19 Hardship related problems’ for each predictor within the Australian born and Refugee Background participants respectively; and p-values represent the significant level within the group.

**Note:** *NA*, Not applicable.

^**b**^ *Chi-square test* conducted to examine the significant variation of ‘Fear or stress associated with COVID-19’ for each predictors within the Australian born women and those from refugee background respectively; and p-values represent the significant level within the group.

#### Fear or stress associated with COVID-19

For women from refugee backgrounds, the significant predictor for fear or stress associated with COVID-19 was number of TE exposures (p = 0.005) and number of finance related stressors (p = 0.001). Amongst Australian born women, a higher number of traumatic events (TE) exposures were significantly (p = 0.008) related to greater fear or stress associated with COVID-19 (Table 3B).

### Predictors of COVID-19 related measures: Adjusted relative risk ratios

Table 4 reports adjusted relative risk ratios (aRRs) for significant predictors for the combined groups of women from the two multiple log-binomial regression models specific to: ‘hardship related to COVID-19’; and ‘fear or stress associated with COVID-19’. Numbers of finance-related stressors was found to significantly associated with material hardship related to COVID-19 and greater fear or stress associated with COVID-19. As compared to ‘no finance-related stressor’, women with three or more stressors had an aRR of 2.14 (95%CI: 1.74–2.49) for hardship related to COVID-19 and an aRR of 1.55 (95%CI: 1.08–2.09) for fear or stress associated COVID-19. The number of traumatic event exposures was found to be a significant predictor of fear or stress associated with COVID-19. Compared with no TE exposures, women with three or more TE exposures had an aRR of 1.70 (95%CI: 1.27–2.14) for fear or stress associated with COVID-19.

**Table 4:** Significant predictors associated with two COVID-19 related questions for the combined sample of Australian born and refugee background participants: Adjusted odds ratios (aORs) with 95% confidence interval (CI) from Stepwise Multiple Logistic Regression Analysis (n = 650).

^d^ Significant predictors	Hardship related to COVID-19^e^	Fear or stress associated with COVID-19^e^
	aOR (95% CI)	aOR (95% CI)
**Country of Birth** ^**f**^		
Australian born women (reference category)	1.00	1.00
Refugee background women	1.61 (1.14–2.27)**	2.06 (1.45–2.93)**
**Number of traumatic events**		
0 (reference category)	NS	1.00
1–2		1.80 (1.21–2.66)**
3 and more		2.33 (1.41–3.83)**
**Number of finance related stressors**		
0 (reference category)	1.00	1.00
1–2	1.82 (1.12–2.96)*	1.59 (0.94–2.70)
3 and more	4.15 (2.53–6.80)**	1.90 (1.11–3.27)*

^**d**^ All the potential predictors (used in bivariate analysis: Table 3A and 3B) are included in the stepwise multiple logistic regression analysis for the combined sample of Australian born and Refugee background participants. Predictors included in the stepwise logistic regression are: Age, Highest level of educational attainment, Women’s employment, Men’s (partners) employment, Ever been Homeless, Number of traumatic events exposures, Number of friends/family members who can be relied on for serious problems and Number of finance related stressors.

^**f**^ In addition to these potential predictors all multiple logistic regression models were adjusted for country of birth: Australian born women (coded as ‘0’; assigned as reference category) and women born in conflict-affected countries (coded as ‘1’).

^**e**^ The outcome measures for multiple logistic regression model was coded as, 0 = no problem at all and 1 = a problem/a very serious problem).

NS, Not found significant in stepwise multiple logistic regression analysis.

* aORs are significant at p<0.05

** aORs are significant at p<0.01.

### All predictors–Women from refugee background compared with Australian born women

Findings also revealed that after adjusting for all potential predictors, women from refugee background have 1.32 times (95%CI: 1.10–1.60) more risk of having COVID-19 related hardship and 1.41 times (95%CI: 1.23–1.61) greater risk of experiencing fear or stress associated with COVID-19 as compared to Australian born women.

### Association of COVID-19 related problems with common mental disorders (CMDs)

The association of the two COVID-19 related measures with CMDs: Major Depressive Disorder (MDD), Post-Traumatic Stress Disorder (PTSD), Persistent Complicated Bereavement Disorder (PCBD), PanicDisorder (PD), and Separation Anxiety Disorder (SEPAD) are presented in Table 5.

**Table 5:** Association of COVID-19 related difficulties with prevalence (%) of Common mental disorders (CMDs) and Relative Risk (RR) with 95% Confidence Interval (95% CI) for Australian born and refugee background participants.

	*Total number of women*	Common Mental Disorders				
		MDD	PTSD	PD	SEPAD	PCBD
COVID-19 related difficulties		Prevalence % (n)	Prevalence % (n)	Prevalence % (n)	Prevalence % (n)	Prevalence % (n)
**Australian born women**						
***All***	*311*	*13*.*5 (42)*	*5*.*1 (16)*	*16*.*1 (50)*	*12*.*9 (40)*	*2*.*9 (9)*
**Hardship related to COVID-19**						
No problem at all	210	11.9 (25)	4.3 (9)	13.3 (28)	11.0 (23)	1.9 (4)
A problem/very serious problem	101	16.8 (17)	6.9 (7)	21.8 (22)	16.8 (17)	5.0 (5)
*Test statistic χ* ^*2*^ *(df) and p-values*		*χ*^*2*^*(1) = 1*.*41; 0*.*234*	*χ*^*2*^*(1) = 0*.*98; 0*.*323*	*χ*^*2*^*(1) = 3*.*57; 0*.*057*	*χ*^*2*^*(1) = 2*.*10; 0*.*147*	*χ*^*2*^*(1) = 2*.*25; 0*.*134*
**RR (95%CI) [ref. cat. no problem]**		1.16 (0.89–1.50)	1.21 (0.78–1.88)	1.24 (0.96–1.61)	1.20 (0.91–1.58)	1.53 (0.74–3.20)
**Fear or stress associated with COVID-19**						
No problem at all	164	9.1 (15)	3.7 (6)	11.6 (19)	8.5 (14)	1.80 (3)
A problem/very serious problem	147	18.4 (27)	6.8 (10)	21.1 (31)	17.7 (26)	4.1 (6)
*Test statistic χ* ^*2*^ *(df) and p-values*		*χ*^*2*^*(1) = 5*.*64; 0*.*018*	*χ*^*2*^*(1) = 1*.*57; 0*.*210*	*χ*^*2*^*(1) = 5*.*18; 0*.*023*	*χ*^*2*^*(1) = 5*.*79; 0*.*016*	*χ*^*2*^*(1) = 1*.*40; 0*.*237*
**RR (95%CI) [ref. cat. no problem]**		1.55 (1.02–2.36)*	1.43 (0.75–2.71)	1.46 (1.01–2.12)*	1.58 (1.02–2.44)*	1.60 (0.63–4.05)
**Women from Refugee Background**						
***All***	*339*	*19*.*8 (67)↑*	*9*.*7 (33)↑*	*5*.*0 (17)↓*	*19*.*8 (67)↑*	*6*.*5 (22)↑*
**Hardship related to COVID-19**						
No problem at all	179	15.1 (27)	8.9.(16)	2.8 (5)	16.2 (29)	5.6 (10)
A problem/very serious problem	160	25.0 (40)	10.6 (17)	7.5 (12)	23.8 (38)	7.5 (12)
*Test statistic χ* ^*2*^ *(df) and p-values*		*χ*^*2*^*(1) = 5*.*23; 0*.*022*	*χ*^*2*^*(1) = 0*.*27; 0*.*601*	*χ*^*2*^*(1) = 3*.*92; 0*.*047*	*χ*^*2*^*(1) = 3*.*03; 0*.*081*	*χ*^*2*^*(1) = 0*.*51; 0*.*475*
**RR (95%CI) [ref. cat. no problem]**		1.39 (1.02–1.89)*	1.10 (0.76–1.58)	1.84 (0.87–3.86)	1.27 (0.95–1.71)	1.17 (0.73–1.878)
**Fear or stress associated with COVID-19**						
No problem at all	106	12.3 (13)	6.6 (7)	2.6 (3)	15.1 (16)	1.9 (2)
A problem/very serious problem	233	22.7 (53)	11.2 (26)	6.0 (14)	21.5 (50)	8.6 (20)
*Test statistic χ* ^*2*^ *(df) and p-values*		*χ*^*2*^*(1) = 5*.*10; 0*.*024*	*χ*^*2*^*(1) = 1*.*72; 0*.*190*	*χ*^*2*^*(1) = 1*.*54; 0*.*214*	*χ*^*2*^*(1) = 1*.*88; 0*.*170*	*χ*^*2*^*(1) = 5*.*38; 0*.*020*
**RR (95%CI) [ref. cat. no problem]**		1.74 (1.04–2.90)*	1.53 (0.78–3.01)	1.82 (0.64–5.14)	1.36 (0.86–2.15)	3.62 (0.96–13.67)

**Abbreviations:** *Major Depressive Disorder (MDD)*, *Post-Traumatic Stress Disorder (PTSD)*, *Persistent Complicated Bereavement Disorder (PCBD)*, *Panic Disorder (PD)*, *and Separation Anxiety Disorder (SEPAD)*.

***ref*. *cat*.** *Reference category; ‘No problem’ category used as ‘Reference category’ to estimate Relative Risk (RR) i*.*e*. *the value of RRs>1*.*0 indicates that the risk is relatively higher for those have ‘A problem/very serious problem’ as compared to women ‘No problem category’*.

*RRs are significant at p<0.05.

^**↑**^ Symbol indicates that prevalence for women from refugee background are significantly higher than the women born in Australia

_**↓**_ Symbol indicates that prevalence for women from refugee background are significantly lower than the women born in Australia.

The prevalence of depression was 13.5% for Australian born women and 19.8% for women from refugee background. For women from refugee backgrounds, prevalence of depression was found to be significantly higher for those who had reported any problems in material hardship related (p = 0.022) and fear or stress associated with COVID-19 (p = 0.014). Amongst Australian born women, prevalence of depression was found to be significantly higher for those who reported only fear or stress as a problem associated with COVID-19 (p = 0.018).

Five percent of the Australian born and 9.7% of the women from refugee background met the criteria for PTSD. Amongst both women from refugee backgrounds and Australian born women, the prevalence of PTSD was not statistically higher amongst those who had reported problems in either of the two COVID-19 measures (material hardship or psychological fear and stress related to COVID-19).

One in seven (16.1%) of the Australian born and 5.0% of the women from refugee backgrounds met the PD criteria (p<0.01). In Australian born women, the prevalence of PD was found to be significantly higher for those who reported any problems with fear or stress associated with COVID-19. For women from refugee backgrounds, a significantly higher rate of PD was observed for those who experienced material hardship problems associated with COVID-19 (Table 5).

Prevalence of SEPAD was 19.8% for women from refugee backgrounds and 12.9% for Australian born women. Amongst refugee women, prevalence of SEPAD was not found to be significantly higher amongst those who reported fear or stress related problems, or material hardship associated with COVID-19.

The prevalence of PCBD was 6.5% for women from refugee backgrounds and 2.9% for Australian born women. For women from refugee backgrounds, prevalence of PCBD was found to be significantly higher for those who also reported problems with fear or stress associated with COVID-19 (p = 0.014).

## Discussion

This is the first large systematic study in a high-income country of women who arrived from refugee backgrounds with a comparison group of those born in the country. It is uncommon for a study to use diagnostic measures and to have examined such a comprehensive array of mental disorders, each theoretically likely to be associated with a disaster or life crisis event.

Our study demonstrates that women from refugee backgrounds and Australian born women are experiencing significant rates of CMD during the pandemic. We found that women from refugee backgrounds had a higher a prevalence of MDD, PTSD, SEPAD, and PCBD during COVID-19 when compared with Australian born women. We also found that mental disorders in the women from refugee backgrounds were more often associated with COVID-19-related psychological fear or stress, a condition which was also correlated with higher exposure to prior traumatic events in the bivariate analysis [27]. A non-conforming finding was panic disorder, which was more prevalent in the Australian born women and was associated with fear and stress problems during COVID-19. Both groups (women from refugee background and those born in Australia) experienced associations between mental disorders and material hardship during COVID-19. This finding is consistent with studies demonstrating negative psychological impacts associated with economic factors on people in high income countries during the pandemic [10,45].

COVID-19 stressors (material hardship and fear or stress) were associated with their higher prevalence of CMDs more often in the women from refugee background. It is noteworthy that almost a third (35.1%) of the refugee background women had reported at least one or more finance-related stressors and this rate was 20.6% for Australian born women. This finding suggests that material and psychological problems may be more strongly interrelated for women from refugee backgrounds. For example, for many people from refugee backgrounds, economic adversity during COVID-19 signalled greater psychological stress and potential for mental disorder because prior to arrival poverty was associated with conditions that endangered life. This underscores the need for policy makers and practitioners to understand interrelated mental health risks for refugee women, including exposure to prior trauma as well as experiences of economic adversity [24].

PCBD was significantly higher in the women from refugee backgrounds and compared with women born in Australia, and it was distinctively associated with stress and fear related to COVID-19. We did not inquire about the nature of the loss, which could have been due to war, conflict, disaster, lack of health care or other reasons. People from refugee backgrounds at the time of our study were also more likely to have lost a family member due to COVID-19 than those born in Australia where death rates remained comparably lower than the average in low or middle income countries.Not being able to return home to participate in traditional cultural rituals following death is a compounding factor that may explain unresolved grief.

Women from refugee backgrounds were more affected by PTSD than Australian born women. In both groups PTSD was not related to stress and fear, or material hardship associated with COVID-19. This finding indicates that PTSD was related to pre-pandemic traumatic events where life was threatened, for women from refugee backgrounds these traumas might include the prior experience of war or conflict, including sexual violence [46].

SEPAD was strikingly higher in women from refugee backgrounds compared with Australian born women. Further SEPAD was much higher for both groups than the average 6% documented in general populations [47]. The higher prevalence of SEPAD in women from refugee backgrounds is consistent within a context where there is a higher quantum of mental disorders including anxiety, which is a recognised risk factor for SEPAD, as well as forced separation from family members left in the country of origin [47].

As mentioned, more Australian born women than women from refugee backgrounds reported panic disorder during the COVID-19. We are unable to explain this non-conforming finding, however it is possible that panic is a more Westernised expression of mental distress [48]. It is also noteworthy that fear or stress problems during COVID-19, commonly correlated with mental disorders in the refugee background group, was associated with panic disorder in the Australian born. This association with fear and stress suggests that those with panic disorder may represent and be an indicator of the most psychologically distressed subgroup of the Australian born cohort. This panic-affected group may therefore signify the Australian-born women in most need of mental health interventions during COVID-19.

The common finding of TEs being a vulnerability factor in relation to the impact of COVID related stressors is of practical and theoretical importance. It is of practical importance given that refugee women are known to have experienced a great deal of past trauma both from living through war and conflict and from intimate partner violence. [20] This finding underscores why they should be given proactive support in the community, particularly during a pandemic. Our finding that TEs place women at risk of COVID-related stressors whether refugee or native born is of theoretical importance because exposure to past trauma amongst women has not previously been assessed in studies of mental health in high income countries during pandemic.

### Strengths and limitations

The results are strengthened by the size of the study, its statistically and culturally robust method and high response rate (a total of 1574 pregnant eligible women were listed for interview at baseline and 1335 women were interviewed, that is, a response rate is 96%). [21] We also used diagnostic mental health measures for a comprehensive and germane assessment of mental disorder experienced during a pandemic, as well as 2 specific questions that enquired into material hardship and psychological stress or fear.

Whilst the two COVID-19 questions cover the extrinsic and intrinsic domains identified as problems in other studies, we did not examine specific COVID-19 experiences in detail (such as lockdown, loss of employment, death of a family member or worry about illness). Therefore, there may be some psychosocial factors related to the COVID-19 pandemic that were not considered by participants when we asked them these broad questions.

The analysis is cross-sectional. We cannot claim that the prevalence of mental disorders, although important to report, were uniquely due to COVID-19, even when those disorders were quantitatively associated with specific COVID-19 hardships. It could be, for example, that having an existing mental illness increased the risk for experiencing greater psychosocial problems during COVID-19.

The study is generalisable to women of child-bearing age, which is also the most significantly represented age group being resettled in high income countries.

The findings cannot be generalised to all refugee background women, however our method for recruitment represented three of the largest refugee groups entering Australia and other refugee-receiving countries at the time of the study (Arabic-speaking countries, Sudan, and Tamil speaking Sri Lankan). By limiting the study to these language groups, we also sought to contain the problems of transcultural measurement error and small cell sizes.

### Conclusion

Our study demonstrates that women from refugee backgrounds have distinctive experiences including material problems and psychological stressors that are associated with higher prevalence of mental disorders during COVID-19. They require urgent and specific attention to their social and economic wellbeing and mental health during the pandemic. It is important that if women present with mental distress, a comprehensive and culturally informed assessment is undertaken for mental illness and psychosocial determinants. Assessment for depression, SEPAD and PCBD should be prioritised for women from refugee backgrounds during COVID-19. Interventions need to take an intersectional approach to ensure distinctive factors relevant to being a refugee and a woman are targeted. It is also important to note that Australian women experienced significant prevalence of MDD, PCBD and SEPAD, such that practitioners should be assessing and intervening to support women impacted by these disorders. Future studies should examine common mental disorders, bereavement and separation anxiety disorders amongst Australian born women during the COVID-19 pandemic.

We have identified the association between material hardship and mental disorders in both groups of women, and this underscores the need for governments to recognise and ensure financial wellbeing as a policy priority in the prevention of mental disorder for all women during COVID-19.
