# Correction: COVID-19 stressors and mental health problems amongst women who arrived as refugees and those born in Australia

**DOI:** 10.1371/journal.pgph.0002455

**Published:** 2023-09-27

**Authors:** Susan J. Rees, Mohammed Mohsin, Alvin Kuowei Tay, Batool Moussa, Louis Klein, Nawal Nadar, Fatima Hussain, Yalini Krishna, Batoul Khalil, Mariam Yousif, Derrick Silove, Jane Fisher

The third author’s name is cited incorrectly. The correct citation is: Rees SJ, Mohsin M, Tay AK, Moussa B, Klein L, Nadar N, et al. (2023) COVID-19 stressors and mental health problems amongst women who arrived as refugees and those born in Australia. PLOS Glob Public Health 3(7): e0002073. https://doi.org/10.1371/journal.pgph.0002073
