# Correction: COVID-19 stressors and mental health problems amongst women who arrived as refugees and those born in Australia

**DOI:** 10.1371/journal.pgph.0002666

**Published:** 2023-11-21

**Authors:** Susan J. Rees, Mohammed Mohsin, Alvin Kuowei Tay, Batool Moussa, Louis Klein, Nawal Nadar, Fatima Hussain, Yalini Krishna, Batoul Khalil, Mariam Yousif, Derrick Silove, Jane Fisher

There are errors in Table 4. Please see corrected Table 4, using aRRs instead of aORs, below.

**Table 4:** Significant predictors associated with two COVID-19 related questions for combined sample of Australian born women and Women from Refugee Background: Adjusted relative risk ratios (aRRs) with 95% confidence interval (CI) from multiple log-binomial regression (n = 650).

d Significant predictors	Hardship related to COVID-19^e^	Fear or stress associated with COVID-19^e^
	aRR (95% CI)	aRR (95% CI)
**Country of Birth** ^** **^ ^f^ ** **		
Australian born women (reference category)	1.00	1.00
Refugee background women	1.32 (1.10–1.60)*	1.41 (1.23–1.61)**
**Ever been homeless **		
No (reference category) [RR]** **	NS	
Yes** **		
**Number of traumatic events **		
0 (reference category)** **	NS	1.00
1–2** **		1.38 (1.12–1.63)**
3 and more** **		1.70 (1.27–2.14)**
**Number of finance related stressors **		
0 (reference category)	1.00	1.00
1–2	1.43 (1.31–1.57)**	1.33 (0.95–1.74)
3 and more	2.14 (1.74–2.49)**	1.55 (1.08–2.09)*

**d** All the potential predictors (used in bivariate analysis: Table 3a, Table 3b) are included in multiple log-binomial regression analysis for combined sample of Australian born women and Refugee background women. Predictors included in multiple log-binomial regression are: Age, Highest level of educational attainment, Women’s employment, Men’s (partners) employment, Ever been Homeless, Number of traumatic events exposures, Number of friends/family members can be relied on for serious problems and Number of finance related stressors.

**e** The outcome measures for multiple log-binomial regression model was coded as, 0 = no problem at all and 1 = a problem/a very serious problem).

**f** In addition to these potential predictors all multiple log-binomial regression models were adjusted for country of birth: Australian born women (coded as ‘0’; assigned as reference category) and women born in conflict-affected countries (coded as ‘1’).

NS, Not found significant in multiple log-binomial regression analysis.

* aRRs are significant at p<0.05;

** aRRs are significant at p<0.01.
